# On Resection of Intestine

**Published:** 1892-09-24

**Authors:** 


### THE PRACTITIONER'S MIRROR AND RETROSPECT.

[*The Editor will be glad to receive offers of co-operation and contributions from members of the profession. All letters should be addressed to* THE EDITOR, THE LODGE, PORCHESTER SQUARE, LONDON, W.]

The "Practitioner's Mirror" is unavoidably diminished in quantity this week, to make room for special "Students'" articles.

### SURGERY.

### ON RESECTION OF INTESTINE.

The chief indications for operation are as follow:—1. When the gut is found to be gangrenous in operations for the relief of intestinal obstruction, *e.g.*, hernia. 2. In some forms of fæcal fistula where milder means have failed. 3. In strictures of the intestine, whether simple or malignant. 4. In irreducible intussusceptions. 5. In occlusions due to peritoneal adhesions where it is impossible to separate the coils of intestine. 6. In extensive lacerations, as after gun-shot wounds, which do not admit of perfect suturing.

In no case should resection be undertaken unless the patient's strength is sufficiently good to stand a prolonged and depressing operation. Symptoms of obstruction are also considered to contra-indicate resection, for in these cases the best results have followed the formation of an artificial anus.

THE OPERATION.

The intestine is drawn out of the abdominal cavity until some four or five inches of healthy gut on each side of the part about to be resected is exposed. This must be carefully laid on a flat sponge or other soft antiseptic material, and then the rest of the peritoneal cavity is to be shut off by the introduction of soft sponges around the protruded intestine. This packing prevents further egress of intestines, and also excludes from the abdominal cavity any of the contents of the intestine that may escape. The intestine is then clamped above and below the diseased portion by one of the various clamps which have been devised for this purpose; or the plan suggested by Dr. Maunsell, of New Zealand, may be used, and which simply consists in wrapping a piece of flat sponge round the intestine, and then transfixing the two ends of the sponge and the intervening mesentery with a safety pin, which is then fastened over the other side of the sponge and bowel. This method is efficient, and easy of application. The bowel is then divided cleanly above and below the diseased portion, and carefully removed along its mesenteric border. In this way the portion to be resected is entirely cut away. All bleeding points should at once be seized with catch forceps. Some operators prefer removing a wedge-shaped piece of mesentery (the base being at the attachment of the portion of bowel to be removed), and then bringing the triangular gap so left together with stitches, after the two ends of divided intestine have been united. If the former plan be adopted—and this is preferred by some because fewer vessels are divided—the omentum must be brought together by a continuous stitch, so that as the ends of the divided intestine are approximated the omentum when sutured stands out at right angles to the longitudinal direction of the gut. The two ends of intestine, after being carefully cleansed, are then approximated and sutured together by one of the various methods described below. This being accomplished the clamps are removed, the bowel returned to the abdominal cavity, and the incision in the parietes closed.

THE DIFFERENT MODES OF UNITING THE INTESTINE AFTER RESECTION.

For this purpose the best needle is an ordinary round sewing needle, which does less damage than the triangular surgical needle, and the best uniting material is fine dentist's silk or very fine silkworm gut. Upon the various kinds of suture (which are very numerous) employed we cannot now enter, but must content ourselves with a brief description of the manner in which the divided portion of intestine may be restored to their continuity.

1. With Circular Suturing of the Divided Portions.—If it be intended to place a double row of stitches, the mucous surfaces of the proximal and distal portions of the gut are first united, and then the serous surfaces are brought together by means of an interrupted suture such as Lembert's. A continuous stitch must not be used, as it would become loose when the bowel contracted. The weakest point is along the line of attachment of the mesentery, and here, therefore, the greatest care is required in the application of the sutures. This operation is apt to be tedious, but it is somewhat facilitated by the plan (suggested by Greig Smith) of raising the peritoneal surface near the divided edge into a transverse fold by placing a Halstead's plain quilt suture on either side of each portion of gut, and making traction on the threads.

2. By Lateral Implantation.—In cases where it is necessary to unite portions of intestine of unequal size, Billroth suggested that the end of the larger segment should be closed completely by invaginating the margins and bringing the peritoneal surfaces together by a continuous stitch. A slit is then made in the side of this piece of intestine—opposite to the mesenteric attachment, and in the long axis of the gut—proportional to the calibre of the smaller portion, the divided end of which is then placed in the slit and carefully united there by suture in such a manner that the edge of the slit is turned in, and so the peritoneal surface of the larger portion is attached to that of the smaller segment.

Senn has modified this method by placing a soft rubber ring inside the smaller piece of intestine, to the lower margin of which it is fastened by a continuous catgut suture. The band is about half an inch wide, and when fixed in position facilitates invagination by causing the gut to take a tapering form. The upper portion of the ring and all the coats of the intestine are then pierced by two catgut sutures—one near the mesenteric border and one on the opposite side—and by these is attached to the invaginated slit in the larger portion of the bowel by passing through its peritoneal and muscular coats at points corresponding to the position of the sutures in the smaller segment. The rubber ring is subsequently discharged.

3. Intestinal Anastomosis by Senn's Method, with Decalcified Bone Plates.—These plates are prepared by making transverse sections of an ox's tibia or femur, about a quarter of an inch thick, and decalcifying them in a 10 per cent. solution of hydrochloric acid. They are oval in shape about two and a half inches long, and one inch wide, having a central aperture around which are four small perforations. Before use they can be pared to the appropriate size with a penknife, and should be threaded with silk or catgut. "The threads or sutures are attached by threading two fine sewing needles, each with a piece of aseptic silk twenty-four inches in length, which are tied together; the knots become the ends of the end or apposition sutures, while the middle of such thread holds the needle and becomes the terminal part of the lateral or fixation sutures. The fastening of the threads upon the plate is done by the lock-stitch by another thread passing through the perforations in the shape of a loop and fastened at the back."

After resection, the free margin of each portion of the intestine is folded in, and then completely closed by a continuous stitch passing through the peritoneal and muscular coats. An incision is then made in each portion of the gut on the side furthest from the mesenteric attachment, in the direction of its long axis, and large enough to admit one of the bone plates easily. The bone plate having been passed into the intestine, and then arranged so that the hole in the plate more or less corresponds to the incision in the gut, the needles attached to the fixation sutures are passed, one on each side, through all the coats of the intestine, and close to the margin of the opening made in the gut. The apposition sutures hang out of the angles of the wound. A similar procedure is then adopted with the other portion of intestine. The two parts of the intestine which are to be united are then approximated so that the two wounds, supported on the inner side by the bone plates, exactly correspond. The adjacent peritoneal surfaces may be scarified with a needle.

"The sutures are now ready for tying together, but before this is done the serous surfaces along the posterior margins of the plates may be united by a few points of suture. After this has been done the posterior pair of fixation sutures is tied with sufficient firmness to approximate, but not to compress the parts between them. The sutures are always to be tied in a square knot, so as to prevent slipping of the knot. Next the pair of end or approximation sutures away from the operator is tied, and when this has been done the opposite pair is tied. All the sutures are cut short as soon as they are tied. The last sutures to be tied are the remaining pair of fixation sutures; and while these are being tightened the margins of the bowel are inverted between the plates with a director or probe. The cut ends of the last knot are pushed with a probe towards the opening. Approximation has now been completed, and all that is left is to reinforce the action of the plates by suturing the serous surfaces over the anterior margins of the plates by a few stitches of the continued suture."

Sachs has proposed the use of one bone plate shaped like a large cuff stud, with a hole through the centre. This is inserted into the incision, each portion of intestine to be approximated, and sutures are then applied to keep the parts firmly together. More recently Dawbarn has replaced the decalcified bone plates of Senn by discs of raw potato. The method of Senn admits of varied application to different parts of the intestine, but it has the disadvantages that the pressure of the plates may cause gangrene of the intervening gut, and that intestinal contents may possibly escape along the path of the threads which perforate all the coats of the intestine.

4. Invagination Method of Dr. Maunsell.—In this procedure, which is somewhat complicated, but fairly easy of performance, that portion of gut which is of smaller calibre is invaginated into that of larger calibre by being dragged through a hole made for that purpose in the side of the more dilated intestine. The two divided portions of intestine are brought together so that the cut ends face each other. They are then fixed together by two temporary sutures passing through all the coats of each portion of bowel, one suture being near the mesenteric border, and the other on the side farthest from the mesenteric attachment. The ends of these sutures are left long. Maunsell considers these very important: "They secure the complete peritoneal covering of the mesenteric attachment of both segments of the gut, help to maintain the proper relative position and accurate co-adaptation of the two cut ends, and facilitate their subsequent invagination through the opening made in the larger segment of gut."

A hole is now cut in the more dilated portion of intestine by pinching up a fold with the finger and thumb and then cutting it off with a scalpel. It should be placed on that part of the intestine farthest from the mesentery, should be about one and a-half inches long in the long axis of the gut, and should commence about one inch from the cut end. Through this hole the long ends of the temporary sutures are now passed, when by making traction upon them the two divided ends of intestine, are invaginated into the larger portion of intestine, and brought out through the hole which has been made in the side of it. "While an assistant holds the ends of the temporary sutures, the surgeon passes a long, fine straight needle, armed with a stout horsehair or very fine silkworm gut, through both sides of the bowel, taking a good grip (quarter of an inch) of all the coats. The suture is then hooked up from the centre of the invaginated gut, divided and tied on both sides. In this way twenty sutures can be rapidly placed in position with the passages of the needle. The temporary sutures are now cut off short, and the sutured ends painted with Woelfler's mixture of alcohol, glycerine, and colophony, and blown over with iodoform, the same that he applies to the surface of the raw stump after removal of the tongue." The suturing being completed, traction is then made upon the smaller segment of intestine, so as to partly reduce its invagination into the larger segment. The edges of the longitudinal slit are then well turned in and brought together with a continuous suture and painted with Woelfler's mixture and iodoform.

It has been shown that a slight diminution in the lumen of the bowel—as in the closure of the longitudinal slit referred to—is not productive of any mischief. "By this simple device, the perfect union by suture of a complete transverse section of the bowel, with its circumferential peritoneal surfaces in exact position, and all the knots of the sutures on the inside, can be accomplished." The process takes about ten minutes, which is only about half the time required for "lateral anastomous." This is a great advantage, and moreover, the risks of gangrene, &c., which are attendant on the use of bone plates are avoided. Even with these favourable points, there are many cases where it would be far less dangerous to complete the operation of enterectomy by the formation of an artificial anus, to be subsequently dealt with, than to adopt any method of intestinal anastomosis. This is notably the case where there are symptoms of obstruction, where it is obvious that the formation of a false anus would at once relieve the symptoms.

The great danger in all the methods is the long time taken up by the operation. Most of the above operations can be applied to the various parts of the intestinal tract, but we cannot enter now upon such modifications as may be necessary in the different regions.

### After Treatment.

The patient should be kept lying on his back, with the knees bent over a pillow. Vomiting should be checked with warm water, as ice is apt to cause abdominal pain. A hypodermic injection of morphia is generally necessary. Nutrient enemata may be given for the first twenty-four hours, and then the patient may be fed upon peptonized milk and beef tea for a week, during which time absolute quietness must be enjoined. The diet may then be gradually improved. The bowels should, if possible, be allowed to act naturally for the first time. Glycerine enemata (when necessary) are best. Much relief is afforded by the occasional passage of a rectal tube.

